# Nanoporous Metal Papers for Scalable Hierarchical Electrode

**DOI:** 10.1002/advs.201500086

**Published:** 2015-06-05

**Authors:** Takeshi Fujita, Yasuhiro Kanoko, Yoshikazu Ito, Luyang Chen, Akihiko Hirata, Hamzeh Kashani, Osamu Iwatsu, Mingwei Chen

**Affiliations:** ^1^ WPI Advanced Institute for Materials ResearchTohoku University2‐1‐1 KatahiraAoba‐kuSendai, Miyagi980‐8577Japan; ^2^ PRESTOJapan Science and Technology AgencySaitama332‐0012Japan; ^3^ Taisei‐Kogyo Co. Ltd26‐1 Ikeda‐kitaNeyagawaOsaka572‐0073Japan; ^4^ State Key Laboratory of Metal Matrix CompositesSchool of Materials Science and EngineeringShanghai Jiao Tong UniversityShanghai200030China

**Keywords:** dealloying, nanoporous metal, oxygen evolution reaction, structural hierarchy, supercapacitor

## Abstract

**Nanoporous metals similar to paper in form** are developed using Japanese washi paper as a template to create hierarchical porous electrodes. This method is used to create a trimodal ­nanoporous Au electrode, as a well as a hierarchical NiMn electrode that achieves high electrochemical capacitance and a rapid rate of oxygen evolution.

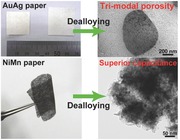

Advanced nanostructured electrodes are a vital component to creating environmentally friendly energy applications such as supercapacitors, batteries, and electrochemical catalyst/conversion technologies.^1^, ^2^, ^3^, ^4^, ^5^, ^6^ 3D nanostructures offering both a high electron conductivity and surface area are required for many such applications, as they allow unimpeded transfer of electrons and ions. This has led to significant attention being given to the creation of nanoporous metals through dealloying, as this provides both a bicontinuous metal structure for electron transport and a catalytically active surface for ion transfer.^7^, ^8^, ^9^, ^10^ Since nanopores (<1 μm) impede mass transport, but micropores (>1 μm) can promote fast mass exchange, a hierarchical structure with pores of different sizes has long been sought as a means of providing smooth mass transport in combination with a large surface area.^11^, ^12^ Some progress has been made toward such a hierarchical (bimodal) porous structure through two‐step dealloying strategies such as dealloying/plating/redealloying^11^ and dealloying/annealing/redealloying,^12^, ^13^, ^14^ but these require multiple electrochemical processes that are too costly and time‐consuming for commercial‐scale production. A bimodal nanoporous structure can also be fabricated by dealloying two‐phase precursors,^15^, ^16^, ^17^ but this strategy limits the metals that can be used.

In this study, an innovative combination of advanced powder metallurgy and dealloying is used to create hierarchical porous electrodes of Au–Ag and Ni–Mn using Japanese “Washi” paper as a template. This is demonstrated to produce a bimodal porous structure from metallized papers via one‐step dealloying, while a first‐ever trimodal porous structure is achieved through the two‐step dealloying of Au–Ag. From an economic point of view, the hierarchical nanoporous NiMn created by one‐step dealloying provides superior areal capacitance with long‐lived cyclability, making it well suited for use as a high‐performance oxygen evolution reaction (OER) electrode. Not only is this method suitable for mass production, but it can also be easily applied to other alloy systems to potentially create hierarchical porous electrodes suitable for such applications as sensing devices, catalysis, or energy storage and conversion.

The present strategy is illustrated in **Figure** **1**a, in which pure metal and alloy powders are first prepared and collected by water atomization and filtering. A template material consisting of cellulosic fibers <50 μm in diameter, namely Japanese “Washi” paper, is then infiltrated with a slurry of metal powder (<5 μm) and water‐soluble binder; capillary action helps to ensure its entire surface is covered. During sintering at high temperature, the template material and carbon‐based binder are decomposed (this process is referred to as “debinding” in powder metallurgy,^18^ while the metal powder remaining becomes consolidated to form a sheet. If different pure metal powders are used in the slurry, e.g., Au and Ag, then a solid‐solution alloy can be created. This sintering technique is applicable to most metal powders, and can be used for net‐shape mass production of ultrathin microporous metal sheets.^19^ At that stage, the microscale porosity can be physically defined by the sintered porous texture. Subsequent one‐step dealloying allows the less noble elements to be etched away, meaning that nanoscale pores can be created in metal sheets with preexisting microscale porosity. Details of this are provided in the Supporting Information.

**Figure 1:**
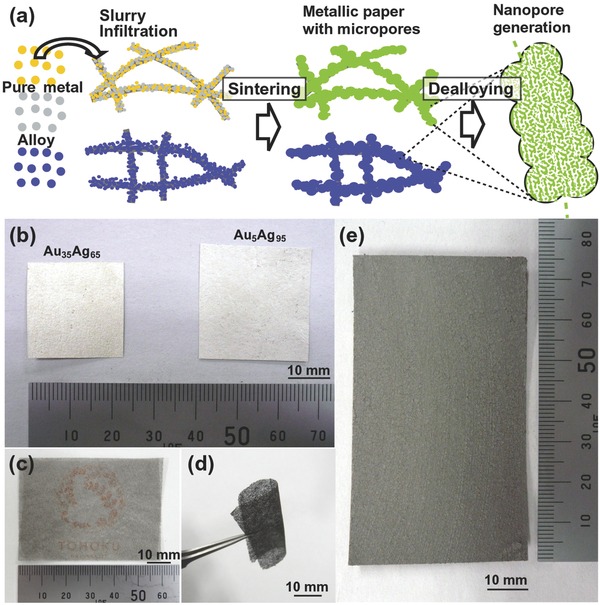
a) Schematic showing the creation of a hierarchical porous structure by sintering a slurry of metal powder and water‐soluble binder on a paper sheet. Appearance of b) ≈100 μm thick Au_35_Ag_65_ and Au_5_Ag_95_ alloy sheets, c) 50 μm thick Ni_30_Mn_70_ alloy sheet (the logo of Tohoku University is on the back), d) bendable thin Ni_30_Mn_70_ alloy sheet, and e) ≈400 μm thick Ni_30_Mn_70_ alloy sheet produced using the method outlined in (a).

Figure 1b shows the appearance of AuAg alloy (Au_35_Ag_65_, Au_5_Ag_95_ (at%)) prior to alloying, while Figure 1c–e shows both thin and thick Ni_30_Mn_70_ alloy sheets. The Au_35_Ag_65_ sheet demonstrates the bimodal porous structure that is possible with one‐step dealloying, while the Au_5_Ag_95_ is believed to be the first example of a trimodal porous structure created through two‐step dealloying.^12^, ^13^, ^14^ The thin NiMn alloy sheet was sufficiently transparent to see the logo of Tohoku University printed on its reverse side, with the high degree of mechanical flexibility retained after dealloying demonstrated by tweezers in Figure 1d. To confirm the mechanical stability, tensile and full bending tests were performed (Figure S1, Supporting Information). The homogeneity of the chemical composition and the crystal structure of each metal sheet were confirmed through energy‐dispersive spectroscopy and X‐ray diffraction (Figures S2−S4 in the Supporting Information).

**Figure** **2**a–c shows the bimodal structure of the Au_35_Ag_65_ sheet created by one‐step dealloying at room temperature in concentrated HNO_3_ solution, having a surface area of 7.6 m^2^ g^−1^, 40 nm nanopores, and a residual Ag content of ≈7 at%. The trimodal porous structure of Au_5_Ag_95_ created through dealloying/annealing/redealloying^12^, ^13^, ^14^ is shown in Figure 2d–f, in which we see that the shorter dealloying time created numerous ≈10 nm nanopores/ligaments, but still retained ≈75 at% of the Ag. Subsequent annealing at 773 K coarsened these nanopores/ligaments up to 500 nm while still maintaining a high Ag content, while the second dealloying produced nanopores/ligaments in the Au. This ultimately resulted in three different levels of porosity: a large number of initial 10−30 μm micropores, coarse (500 nm) pores generated by heat treatment, and nanopores (20 nm) created by the second dealloying. This gave a total surface area of 17 m^2^ g^−1^ and a final residual Ag content of ≈2 at%. Additional SEM images of the bimodal and trimodal nanoporous Au (NPG) are shown in Figures S5 and S6 in the Supporting Information.

**Figure 2:**
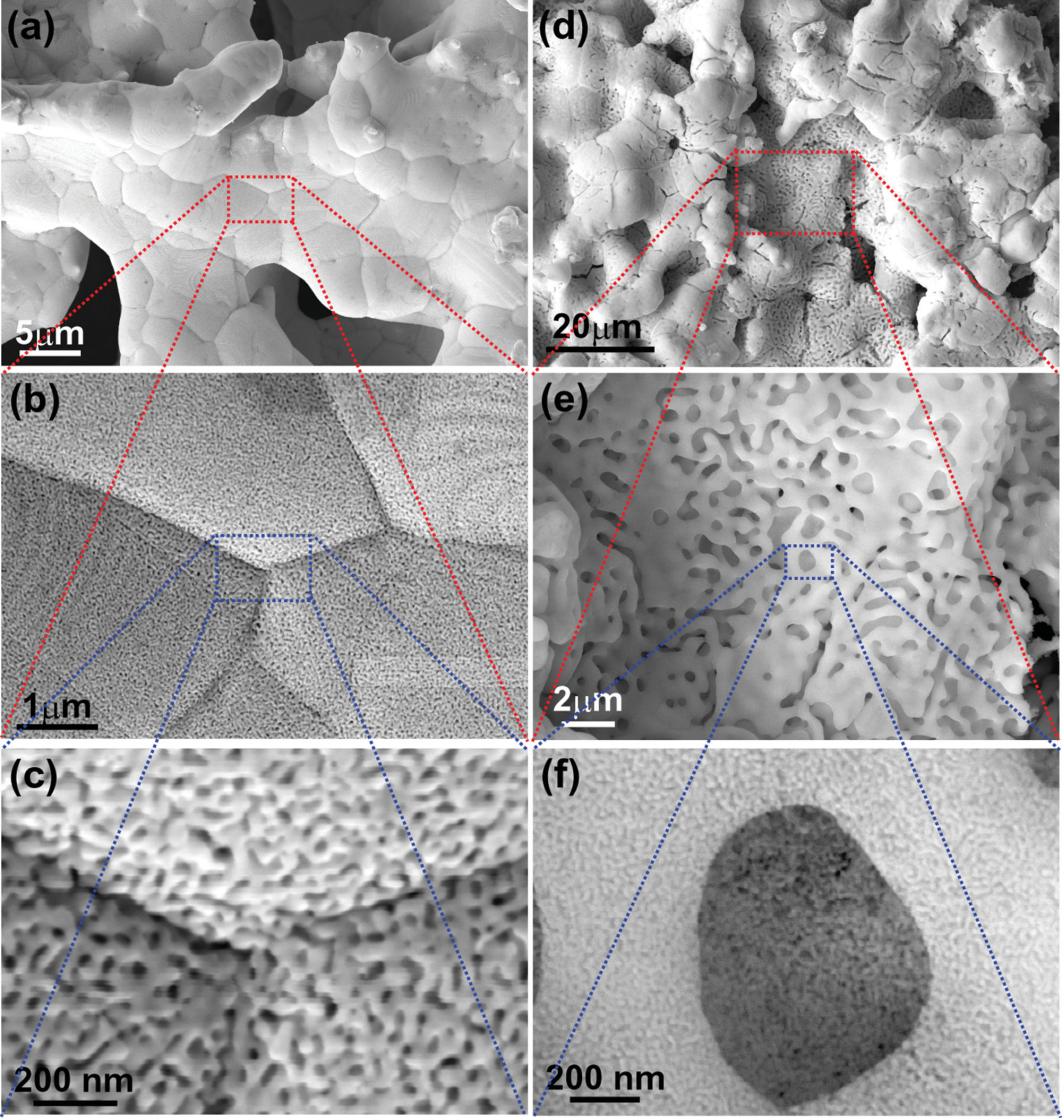
SEM images of a–c) bimodal and d–f) trimodal nanoporous Au at different magnifications. The porosity of the trimodal structure has three different levels: 10 μm initial micropores, ≈500 nm pores coarsened by heat treatment, and ≈20 nm nanopores created by a second dealloying.

To provide a more practical perspective, a cheaper hierarchical nanoporous NiMn structure was fabricated using one‐step dealloying, as shown in Figure S7 (Supporting Information). This resulted in a microporous nanostructure with a residual Mn content of ≈15 at%, but the distinct nanopores seen in NPG were not observed by SEM even at moderate magnification. Subsequent TEM imaging, however, revealed a well‐developed nanosheet structure (**Figure** **3**a). More importantly, the BET surface area increased up to 101 m^2^ g^−1^. This is the highest surface area that has been reported with nanoporous Ni,^13^, ^20^, ^21^ and represents a significant increase from its initial value of 0.9 m^2^ g^−1^ prior to dealloying. The oxide seen to develop on this structure is not usually seen with conventional nanoporous Ni,^20^, ^22^ although this could be explained by the increase in microporosity between ligaments providing more space for structure development. Chemical mapping by TEM energy‐loss electron spectroscopy (EELS) (Figure 3b) and X‐ray photoelectron spectroscopy (Figure S8, Supporting Information) identified these nanostructures as complex oxides such as nickel hydrate Ni(OH)_2_ and manganese oxide, as well as unoxidized Ni. In particular, the residual Mn forms sheet‐like MnO*_x_* oxides during dealloying, as well as Ni(OH)_2_, because the relatively high initial surface area (0.9 m^2^ g^−1^) can promote a stronger oxidation process during dealloying in the case of developed filament‐like nanostructures. This leads to the high surface area (101 m^2^ g^−1^).

**Figure 3:**
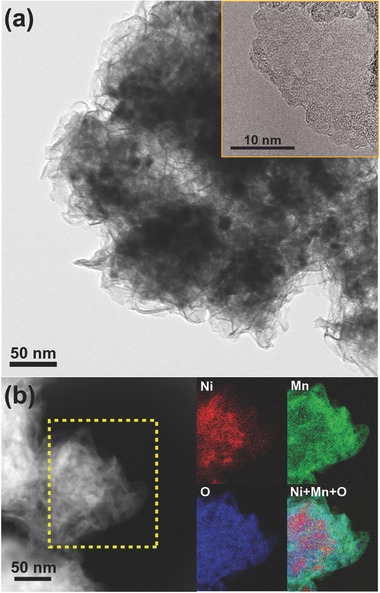
TEM image and EELS chemical map of hierarchical nano­porous Ni. a) Low‐ and high‐magnification images (inset) showing the oxide nanosheet created on the surface. b) STEM image and chemical maps of the selected area showing the distribution of Ni (red), Mn (green), and O (blue).

The nanopore size distributions of the as‐fabricated bimodal, and trimodal NPG, along with the dealloyed hierarchical nanoporous Ni, were evaluated using the Barrett–Joyner–Hallender (BJH) method,^23^ as shown in Figure S9 (Supporting Information). In this figure, the pore distribution of the bimodal NPG exhibits a sharp peak at 45 nm and that of the trimodal NPG possessed a peak at 19 nm with long‐range distribution of more than 100 nm for coarsened pores. This is almost identical to the observed pore sizes in the SEM images. The pore distribution of the dealloyed hierarchical nanoporous Ni exhibits a sharp peak at 10 nm and a small, broadened peak at 100 nm. The micropore size distributions were confirmed using a mercury porosimetry analysis technique, as shown in Figure S10 (Supporting Information), providing evidence for hierarchical pore distribution from the nano‐ to microscale within the samples. In addition, the electron conductance measurements (Figure S11, Supporting Information) of the as‐fabricated bimodal and trimodal NPG, and the dealloyed hierarchical nanoporous Ni, indicate that they exhibit a high electron conductivity that is suitable for electrode applications.

Electrochemical measurement of ≈130 μm thick hierarchical nanoporous Ni was performed in 1 m KOH electrolyte, with **Figure** **4**a showing the typical cyclic voltammetry (CV) curves obtained with scan rates of 1.0–50 mV s^−1^. A pair of well‐defined redox peaks at 0.25 and 0.4 V (vs Ag/AgCl) can be clearly observed in the CV curves, particularly at the lower scan rate, and these are associated with the reversible reactions between Ni^2+^/Ni^3+^ and OH^−^ anions.^24^, ^25^ Electrochemical impedance measurement (Figure S12, Supporting Information) revealed that the electrode exhibits a small semicircle in the high‐frequency region, indicating it has a low charge‐transfer resistance (≈0.2 Ω). In the charge/discharge curves (Figure 4b), the voltage plateaus at around 0.25–0.30 V and the curve trends with *iR* drops are consistent with previous reports,^24^, ^25^ and differ significantly from those of carbon‐based materials for double‐layer capacitance. The specific capacitance calculated from the discharge curves is shown in Figure 4c, with these values per unit of mass being compatible with previously reported values for carbon‐based materials.^26^ In those instances where the value was higher than that of carbon, the areal capacitance (3.7–4.4 F cm^−2^) measured at a discharge current density of 0.04–1 A g^−1^ (1.04–26 mA cm^−2^) was roughly three times greater than that of conventional and oxy‐hydroxide nanoporous Ni (0.7–1.7 F cm^−2^).^13^, ^20^, ^21^ It even proved to be higher than, or similar to, previously reported values for free‐standing pseudocapacitive electrodes such as Co_3_O_4_/MnO_2_ nanowires/nanosheets (0.4–0.71 F cm^−2^),^27^ Co_3_O_4_/NiO core‐shell nanostructure arrays (1.3–2.56 F cm^−2^),^28^ MnO_2_–NiO nanoflakes (0.22–0.4 F cm^−2^),^29^ NiCo_2_O_4_ nanoneedle/Ni forms (0.59–3.12 F cm^−2^),^30^ and 3D ordered nanoporous NiMoO_4_ (2.18–4.25 F cm^−2^).^31^ Moreover, the volume of the electrode minus its pore volume that was estimated from its BET surface area suggests a value of 4.4 F cm^−2^ corresponds to ≈887 F cm^−3^. This highly conductive macroporous metal can, therefore, provide sufficient electrical/ionic conductivity to improve the pseudocapacitive behavior of the Ni(OH)_2_ and MnO*_x_*, as observed in the EELS chemical mapping (Figure 3b). Both of these materials have high theoretical capacitances of 2082 and 1370 F g^−1^, respectively.^24^, ^32^ The capacitance retention of the electrode from its first to 2000th cycle at a current density of 1 A g^−1^ (26 mA cm^−2^) is shown in Figure 4d, in which the areal capacitance interestingly shows a gradual increase up to ≈4.6 F cm^−2^ by the ≈500th cycle. This suggests the activation process increases the number of available active sites, thereby allowing trapped ions to gradually diffuse out, as has been reported for the NiO/Ni(OH)_2_ system.^33^ After 500 cycles, the capacitance decreases slightly down to 85% at the 2000th cycle, but the capacitance after 2000 cycles (3.5 F cm^−2^) is still two times greater than that of conventional and oxy‐hydroxide nanoporous Ni.^13^, ^20^, ^21^ Additionally, the electrode maintains a low charge‐transfer resistance (≈1 Ω) in the high‐frequency region, as determined by electrochemical impedance measurement (Figure S12, Supporting Information). The excellent electrochemical performance of the hierarchical nanoporous Ni can therefore be attributed to: a high capacitance through a large surface area of over 100 m^2^ g^−1^, large micropores that allow the electrolyte to move, and electron transfer from pseudocapacitive Ni(OH)_2_ and MnO*_x_* through the low‐resistance metallic structure (Figure S11, Supporting Information). In order to determine the source of capacitance decay from a microscopic perspective, the TEM images and EELS chemical analysis after 2000 cycles (Figure S13, Supporting Information) are considered. They indicate a minor change in morphology resulting in rod‐like nanostructure formation for Ni(OH)_2_, along with a reduction in MnO_*x*_ content from the initial 15 to 10 at% after 2000 cycles for Mn.

**Figure 4:**
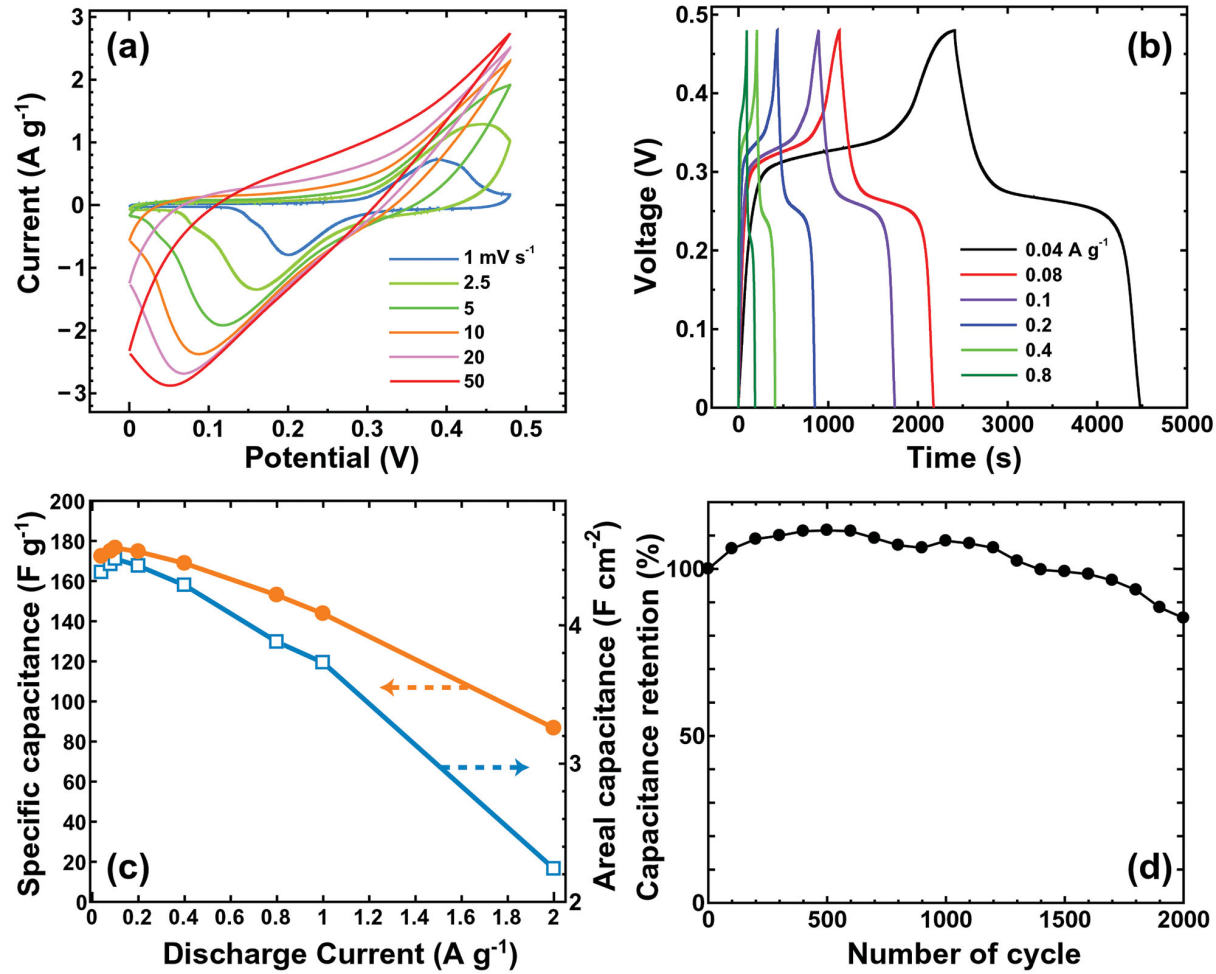
Capacitive performance of the hierarchical nanoporous Ni electrode. a) Cyclic voltammogram curves at different scan rates. b) Charge/discharge curves at different current densities. c) Specific capacitance versus current density. d) Cycling stability during charge/discharge testing at a current density of 1 A g^−1^. The mass of the electrode, including unoxidized nonactive Ni and active materials, has been taken into account.

The OER activity of the hierarchical nanoporous Ni electrode in 1 m KOH electrolyte was also assessed through *iR* corrected CV curves obtained at a scan rate of 1 mV s^−1^, which is shown in **Figure** **5**a, and revealed anodic and cathodic peaks at 390 and 260 mV (vs Ag/AgCl), respectively, at the 1 mV s^−1^ scan rate. A peak separation of 140 mV can also be seen, with this corresponding to the Ni(OH)_2_/NiOOH redox reaction. As shown in the enlarged potential–current curves in Figure 5b, the onset potential of OER for the electrode occurs at 307 mV of overpotential (vs 1.23 V of a reversible hydrogen electrode (RHE)), which is lower than, or similar to, values achieved with solution‐processed MnO*_x_* (514 mV), Ni_0.75_Co_0.25_O*_x_* (312 mV), NiO*_x_* (300 mV), and Ni_0.9_Fe_0.1_O*_x_* (297 mV).^34^ When the OER current density reached 10 mA cm^−2^, the applied overpotential was 337 mV. The electrochemical stability was evaluated by continuous CV cycling at a scan rate of 5 mV s^−1^ (Figure 5c). The pronounced anodic and cathodic peaks after 1000 cycles are representative of the same phenomenon observed in Figure 4d. The slope of the Tafel plots is 54 mV decade^−1^ during the first cycle, and this increases only slightly to 57 mV decade^−1^ by the 1000th cycle. This minimal change indicates a high structural stability against OER and, the values are smaller than in the case of Co_3_O_4_/graphene (67 mV decade^−1^)^35^ and are close to those for ultrathin NiCoFe hydroxide (53 mV decade^−1^).^36^ This combination of shallow Tafel slope, small onset potential, and nanostructure shows greater similarity with α‐Ni(OH)_2_ than β‐Ni(OH)_2_ nanostructures.^37^ The OER for Ni‐based catalysts in alkaline electrolyte involves the following three consecutive elementary steps:^38^

(1) $$\mathrm{NiOOH}+{\mathrm{OH}}^{-}↔\mathrm{NiO}{\left(\mathrm{OH}\right)}_{2}+e^{-}$$

(2) $$\mathrm{NiO}{\left(\mathrm{OH}\right)}_{2}+{\mathrm{2OH}}^{-}↔{\mathrm{NiOO}}_{2}+{\mathrm{2H}}_{2}O+{\mathrm{2e}}^{-}$$

(3) $${\mathrm{NiOO}}_{2}+{\mathrm{OH}}^{-}\to \mathrm{NiOOH}+O_{2}+e^{-}$$

(4) $$\mathrm{Total}\;\mathrm{reaction}:{\mathrm{4OH}}^{-}\to O_{2}+{\mathrm{2H}}_{2}O+{\mathrm{4e}}^{-}$$

**Figure 5:**
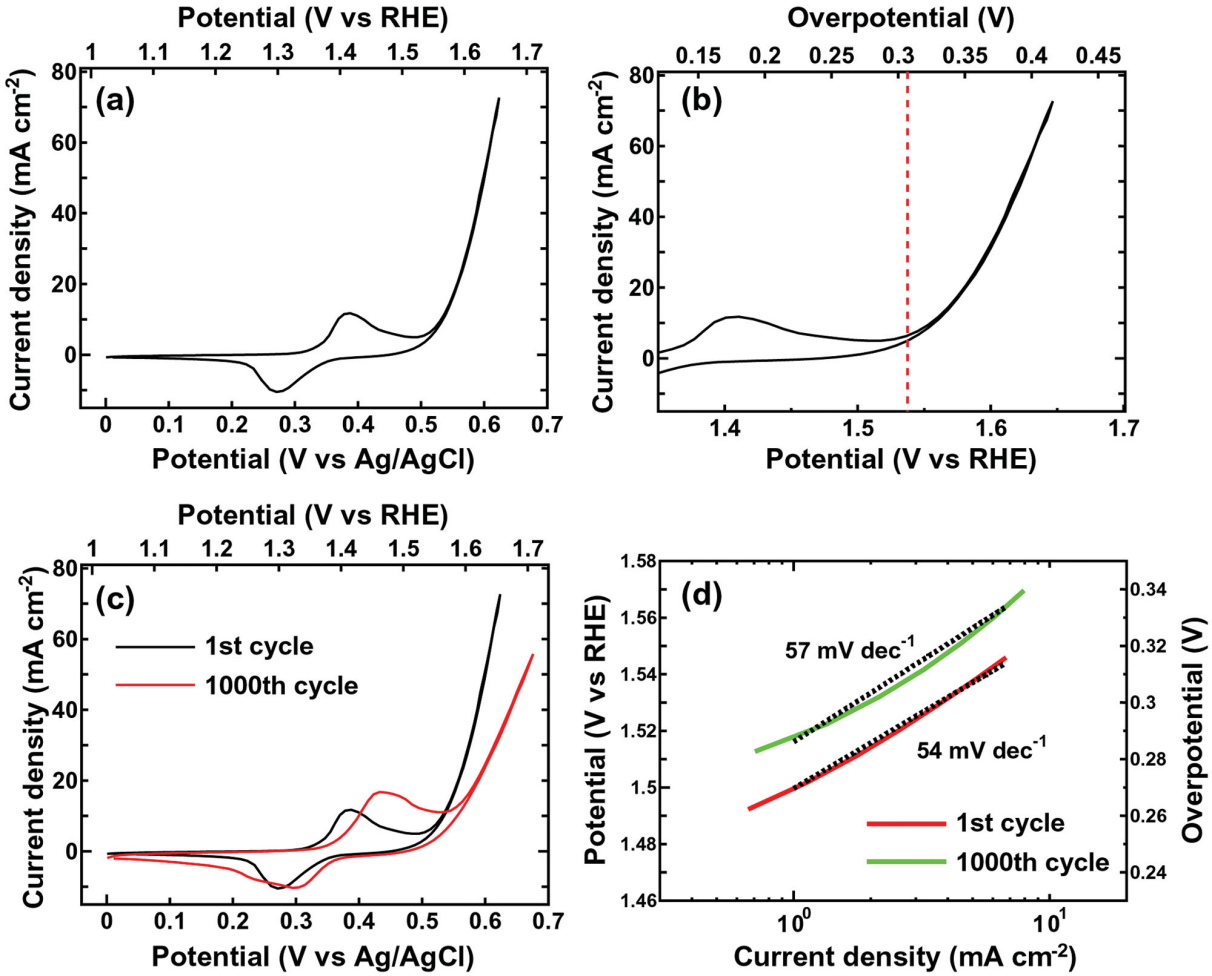
OER performance of the hierarchical nanoporous Ni electrode (all data *iR* corrected). a) CV curve showing the redox reactions at a scan rate of 1 mV s^−1^. b) Enlarged profile showing the onset overpotential at 307 mV (vs 1.23 V of RHE), as indicated by the dotted line. c) CV curves after 1 and 1000 cycles at a scan rate of 1 mV s^−1^. d) Tafel curves of the first and 1000th cycles.

Steps (1) and (2) are reversible and determine the overall OER rate, while step (3) is fast and irreversible. The formation of the NiOO_2_ can be treated as the chemisorption of the O_2_^2−^ ion and determines the underpotential and formation of the O_2_ molecule. It can also explain the small values of the Tafel slope at low current densities.^38^ The OER seems to have a similar mechanism here because the Ni elements are partially oxidized into NiOOH, as evidenced by the oxidation peak at approximately 1.4 V versus RHE. The NiOOH can be further oxidized into NiOO_2_ at a higher potential, and a further electro‐oxidation leads to O_2_ evolution and NiOOH regeneration.

In conclusion, a novel mass‐producible method for making hierarchical porous electrodes has been developed that combines powder metallurgy based on a paper template with subsequent dealloying. This has been demonstrated to be capable of creating both bi‐ and trimodal porous AuAg structures, as a well as a hierarchical nanoporous NiMn electrode that offers a high electrochemical capacitance and OER activity. It should be stressed here that further optimization of these porous structures through tailoring of the fabrication process is undoubtedly necessary to maximize their performance. Nevertheless, the present approach can already be easily scaled up to achieve high outputs of advanced electrodes. We also believe that the strategy presented here can be easily applied more generally to other alloy systems for the development of sensing devices, catalysts, and energy storage/conversion systems for a more sustainable society.

## Supporting information

As a service to our authors and readers, this journal provides supporting information supplied by the authors. Such materials are peer reviewed and may be re‐organized for online delivery, but are not copy‐edited or typeset. Technical support issues arising from supporting information (other than missing files) should be addressed to the authors.

Supplementary
