# Supplementary material for: Nanoporous Metal Papers for Scalable Hierarchical Electrode
Source: Adv Sci (Weinh). 2015 Jun 5;2(8):1500086. doi: 10.1002/advs.201500086 (PMC5115420; doi:10.1002/advs.201500086)
Supplement: Supplementary file 1 — Supplementary [file ADVS-2-0o-s001.pdf]

Copyright WILEY-VCH Verlag GmbH & Co. KGaA, 69469 Weinheim, Germany, 2015.

ADVANCED SCIENCE
Open Access

Supporting Information

for *Adv. Sci.*, DOI: 10.1002/advs. 201500086

Nanoporous Metal Papers for Scalable Hierarchical Electrode

Takeshi Fujita, Yasuhiro Kanoko, Yoshikazu Ito, Luyang Chen, Akihiko Hirata, Hamzeh Kashani, Osamu Iwatsu, and Mingwei Chen*

Copyright WILEY-VCH Verlag GmbH & Co. KGaA, 69469 Weinheim, Germany, 2013.

Supporting Information

Nanoporous Metal Papers for Scalable Hierarchical Electrode

Takeshi Fujita[1,2], Yasuhiro Kanoko[3], Yoshikazu Ito[1], Luyang Chen[1], Akihiko Hirata[1], Hamzeh Kashani[1], Osamu Iwatsu[3], Mingwei Chen[1,4]

[1]WPI Advanced Institute for Materials Research, Tohoku University, 2–1-1 Katahira, Aoba-ku, Sendai 980–8577 (Japan)
[2]PRESTO, Japan Science and Technology Agency, Saitama 332-0012 (Japan)
[3]Taisei-Kogyo Co. Ltd, 26-1 Ikeda-kita, Neyagawa, Osaka 572-0073 (Japan)
[4]State Key Laboratory of Metal Matrix Composites, School of Materials Science and Engineering Shanghai Jiao Tong University, Shanghai 200030 (China)
Electronic mail: tfujita@wpi-aimr.tohoku.ac.jp

1. Materials and Methods

1.1 Preparation of sintered metal using a paper template

Water-atomized micro powders (< 5 μm) of pure gold and silver were purchased from Tanaka Holding Co. Ltd. (http://pro.tanaka.co.jp/en/), while $Ni_{30}Mn_{70}$ micro powders were sourced from Fukuda Metal Foil & Powder Co. Ltd (http://www.fukuda-kyoto.co.jp/00index-e/index.html). The micro powder particle sizes were required to be less than 5 μm to yield alloyed papers with good formability.[S1] In both cases, the water-soluble binder was mixed with carboxymethyl cellulose (CMC) (Sunrose-MAC, Nippon Paper Industries Chemical Division) and water to a CMC/water ratio of 1:9 by weight. A metal slurry was then prepared by mixing the metal powders and binder to a ratio of 1:9 by volume.[S1] The pure gold and silver powders were mixed to give compositions (in mol.%) of $Au_{35}Ag_{65}$ and $Au_{5}Ag_{95}$. To provide a template material for sintering of the metal slurry, traditional Japanese "Washi" paper was purchased from the Awagami Factory in Japan

(http://www.awagami.com/). The final thickness of the alloy could be varied from ~50 to ~400 μm by varying the weight of the paper used from 8 to 60 g m^{-2}. Green compacts were prepared by infiltrating the paper with nine-times its volume in metal slurry. These compacts were then sintered at 1173 K for 2 h in the case of AuAg, or at 1123 K for 2 h for NiMn. An argon atmosphere was used to prevent oxidation.

1.2 Preparation of nanoporous metal

The $Au_{35}Ag_{65}$ alloy was dealloyed in concentrated HNO_3 solution (69 wt%) for 12 h at room temperature to create a bi-modal porous structure. The Au_5Ag_{95} alloy was also dealloyed in concentrated HNO_3 solution (69 wt.%), but for only 1 h at room temperature. After rinsing with water, it was annealed at 773 K for 1 h, and then de-alloyed for a second time for 2 h at room temperature to produce a tri-modal porous structure. The $Ni_{30}Mn_{70}$ alloy was dealloyed in a 1.0 M $(NH_4)_2SO_4$ aqueous solution for 13 hours at 323 K. After dealloying, all samples were rinsed thoroughly with water and ethanol, and then dried under vacuum.

2. Microstructure and properties

2.1 Microstructural characterization

The microstructure of the nanoporous Ni and graphene was characterized using a scanning electron microscope (SEM, JEOL JSM-6700) and transmission electron microscope (TEM, JEOL JEM-2100F) equipped with aberration correctors (CEOS GmbH) for the image- and probe-forming lens systems and an X-ray energy-dispersive spectroscope (EDS) (JED-2300T, JEOL) for chemical composition analysis. SEM analysis was conducted at an accelerating voltage of 15.0 kV for observation and 30.0 kV for EDS mapping. High-resolution TEM and STEM observation was conducted at an accelerating voltage of 200.0 kV, with both Cs correctors optimized for point-to-point resolutions of 1.3 and 1.1 Å for TEM and STEM, respectively. A probe convergence

angle of 29 mrad and HAADF detector with an inner angle greater than 100 mrad was used for HAADF-STEM; the samples having been transferred onto a Cu grid without a uniform carbon support film. The chemical state of each sample was measured using an X-ray photoelectron spectroscope (XPS, AXIS ultra DLD, Shimadzu) with a monochromated AlK_α radiation source. X-ray diffraction (XRD) profiles were obtained using a Rigaku SmartLab X-ray diffractometer with Cu K_α radiation at 40 kV.

2.2. Surface measurements

The nanopore sizes and surface areas of the samples were measured using the Brunauer–Emmett–Teller (BET) and Barrett-Joyner-Hallender (BJH) methods at 77.0 K using a BELSORP-mini II (BEL JAPAN, INC.), which has an upper pore-size limit of 350 nm. The horizontal axis was normalized against the vapor pressure of nitrogen (P_0) at 77.0 K (= 0.101 MPa). The surface area and volume were determined using the BET method, and the micro-scale pore sizes were measured via a mercury porosimetry analysis technique using an Autopore V 9620 (Micromeritics Instrument Corporation, USA). Each sample was first heated at 120 °C under vacuum for 48 h prior to measurement to remove any residual water. The mass of each sample was measured with an ultramicro balance.

2.3. Electrochemical analysis

For measurement of the capacitive performance and oxygen evolution reaction (OER), 1 M KOH was chosen as the electrolyte. The reactivity was measured using saturated calomel electrodes (SCE, BAS Inc.) and Pt electrodes (BAS Inc.) as the reference and counter electrode, respectively. Electrochemical characterization was carried out using a classic three-electrode setup (Iviumstat electrochemical analyzer, Ivium Technology), in which the reference electrode was calibrated with respect to a reversible hydrogen electrode (RHE) using platinum wire as the working and counter

electrode. The specific capacitance (C_s) is calculated from: $C_s = i/[-(\Delta V/\Delta t)m]$, where i is the current, $-\Delta V/\Delta t$ is the slope of the discharge curve after the initial voltage drop and m is the mass of the electrode. The typical electrode mass was ~10 mg. The areal specific capacitance is expressed as:[S2] $C = i\Delta t/S\Delta V$, where i is the current, Δt is the discharge time, ΔV is the voltage range and S is the nominal area of the free-standing electrode. Using this, a Nyquist plot of the nanoporous structure was obtained over a range of 100 kHz to 0.1 Hz at an amplitude of 50 mV.

2.4. Transport properties

The electrical conductances were measured using a 4-probe method in the 270−370-K range. The nanoporous graphene sheets were placed on the SiO_2/Si (SiO_2: 300 nm) substrate and the electrode was fabricated using an Ag epoxy (H20E Epoxy Technology). Each V_+ and V_- electrode was attached in the same manner with the same dimensions. The electrical resistance measurements were performed using the Physical Properties Measurement System (Quantum Design).

2.5. Mechanical test

A dealloyed hierarchical nanoporous Ni sheet specimen with 8-mm length, 3-mm width, and 0.05-mm thickness was tested on a Shimadzu EZ-SX model testing machine at room temperature in air at a strain rate of 0.001 s^{-1}.

3. Mechanical stability of dealloyed hierarchical nanoporous Ni sheet

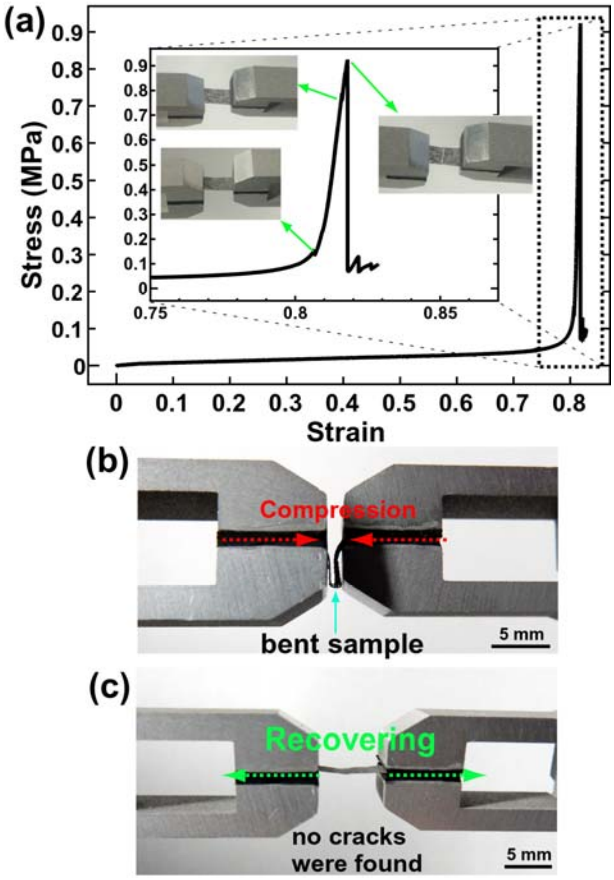

Figure S1. (a) Tensile test of dealloyed hierarchical nanoporous Ni sheet. Before the sample failure, no crack was observed. The crack then formed at the failure point. (b) Compression test, with fully bent sample. (c) Recovered sample after full bending with no crack formation.

4. SEM analysis of AuAg and NiMn before dealloying

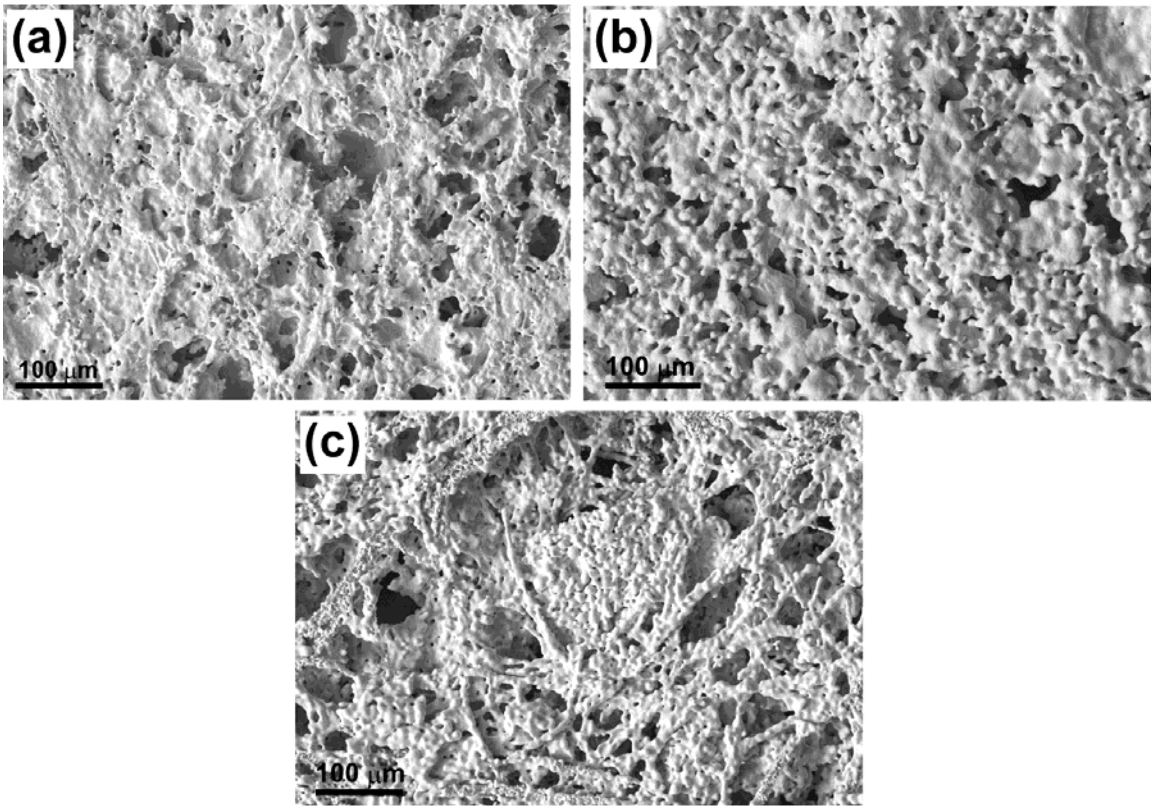

Figure S2. SEM images of (a) $Au_{35}Ag_{65}$, (b) Au_5Ag_{95}, and (c) $Ni_{30}Mn_{70}$ prior to dealloying showing the characteristic microporous texture of the paper template.

5. SEM-EDS analysis of AuAg and NiMn before dealloying

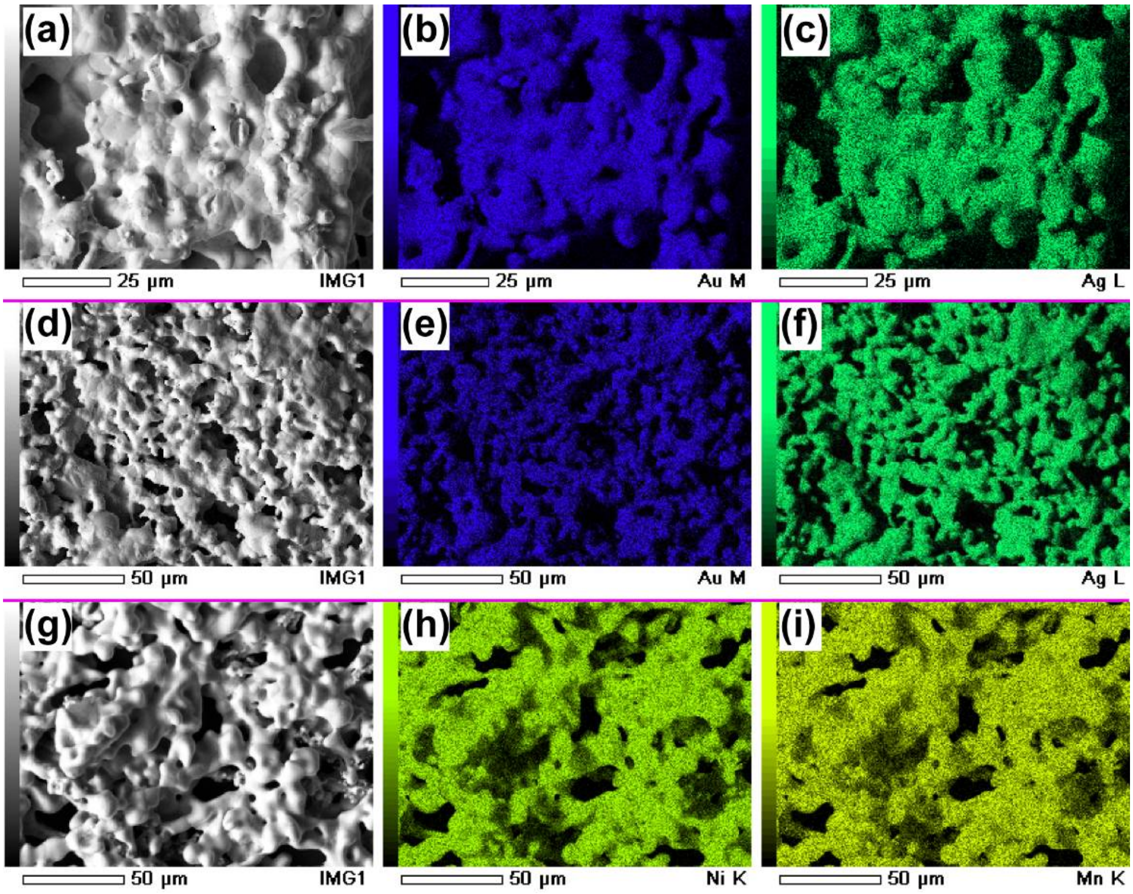

Figure S3. SEM-EDS maps of (a–c) $Au_{35}Ag_{65}$, (d–f) $Au_{5}Ag_{95}$, and (g–i) $Ni_{30}Mn_{70}$ before dealloying. (a,d,g) SEM images, (b,e) Au-M_{α} line, (c,f) Ag-L_{α} line, (h) Ni-K_{α} line, (i) Mn-K_{α} line.

6. XRD analysis of AuAg and NiMn before and after dealloying

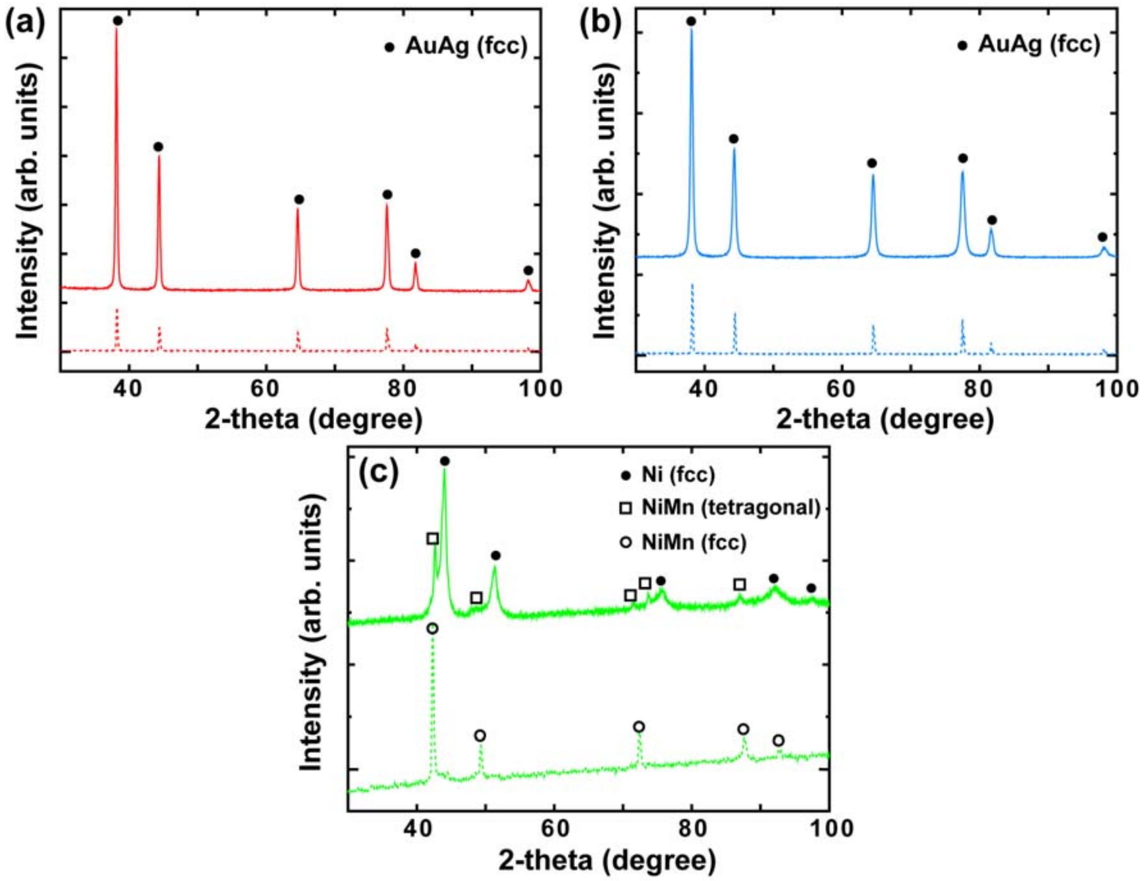

Figure S4. XRD profiles before (solid line) and after (dotted line) dealloying. (a) Bi-modal nanoporous gold and $Au_{35}Ag_{65}$ precursor, (b) tri-modal nanoporous gold and Au_5Ag_{95} precursor, (c) bi-modal nanoporous Ni and $Ni_{30}Ag_{70}$ precursor.

7. Additional SEM images of bi-modal and tri-modal nanoporous Au

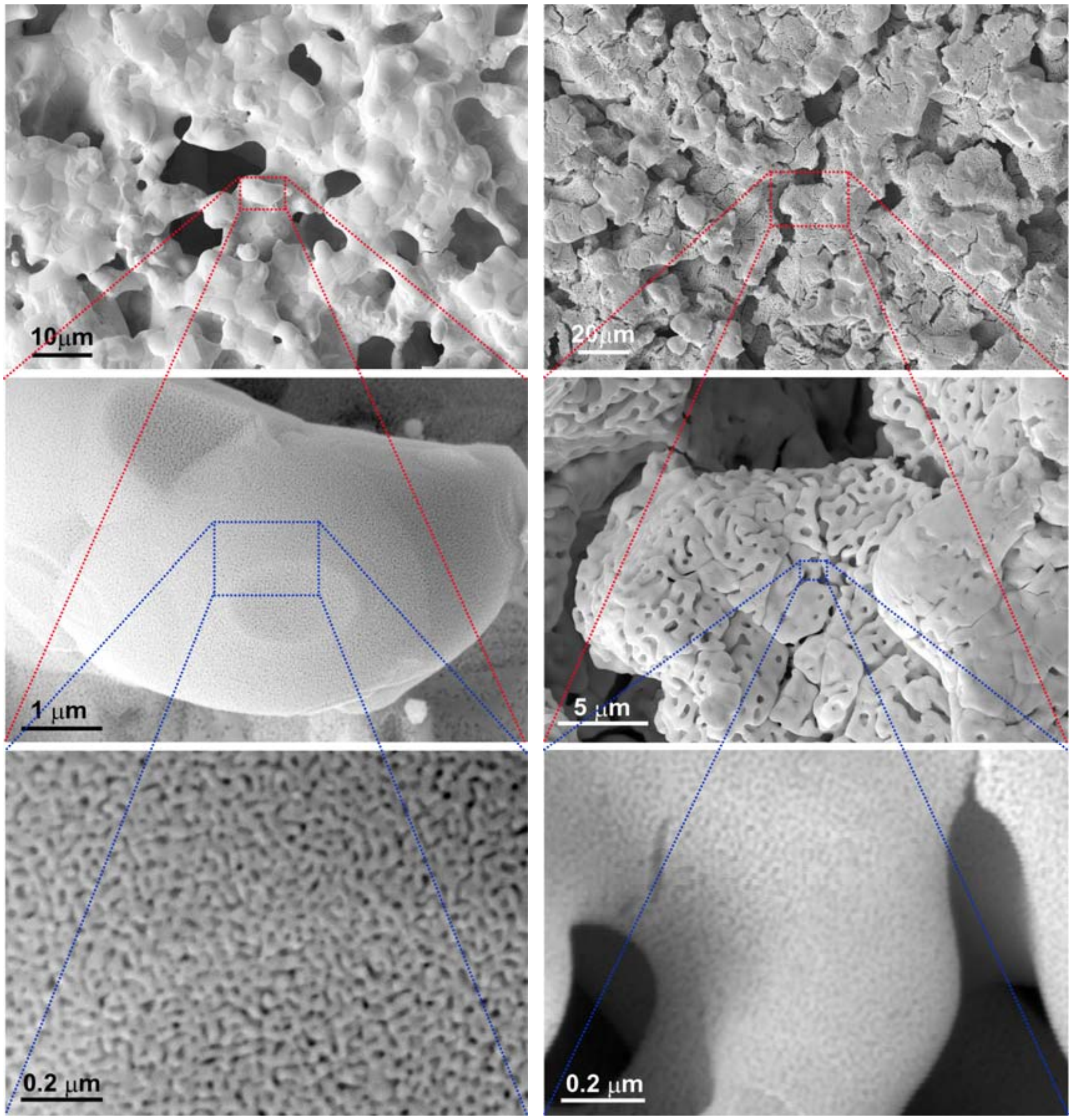

Figure S5. SEM images of (left) bi-modal and (right) tri-modal nanoporous Au at different magnifications.

8. Additional SEM images of tri-modal nanoporous Au

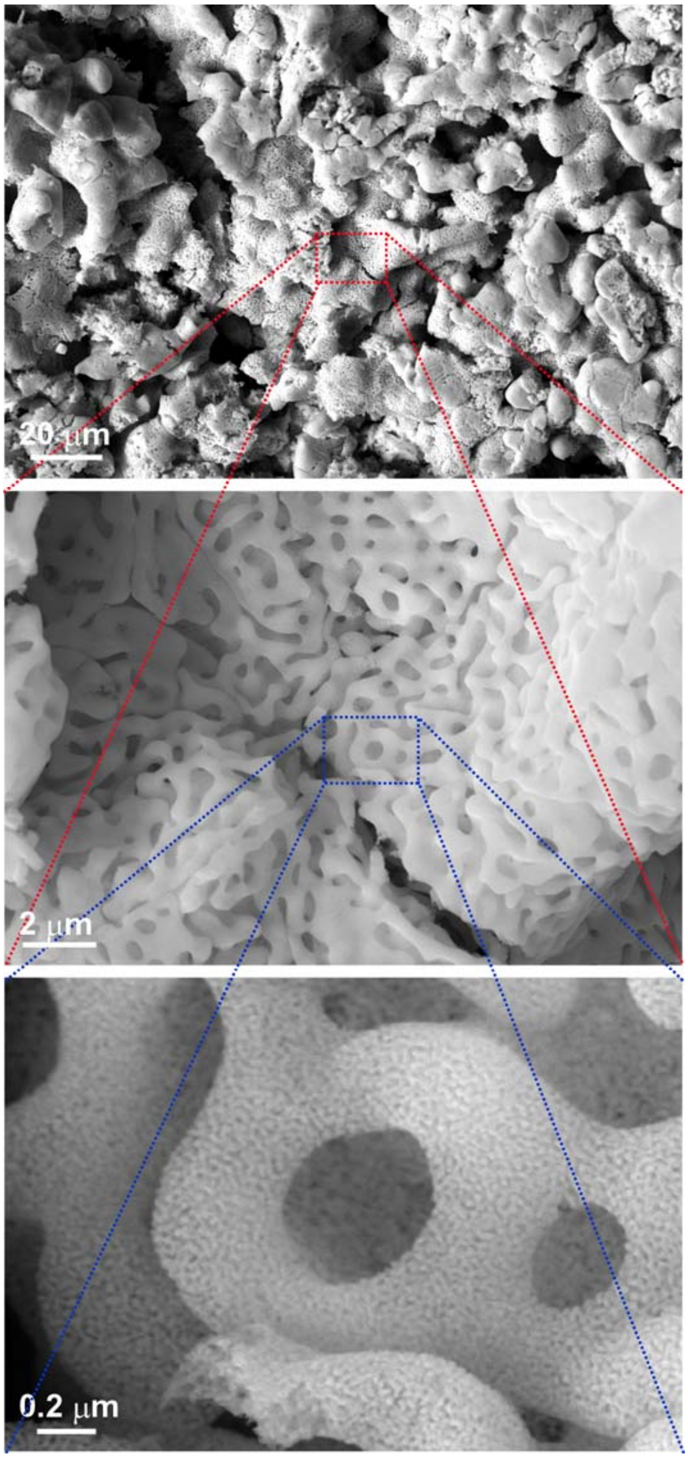

Figure S6. SEM images of tri-modal nanoporous Au at different magnifications.

9. SEM analysis of hierarchical nanoporous Ni

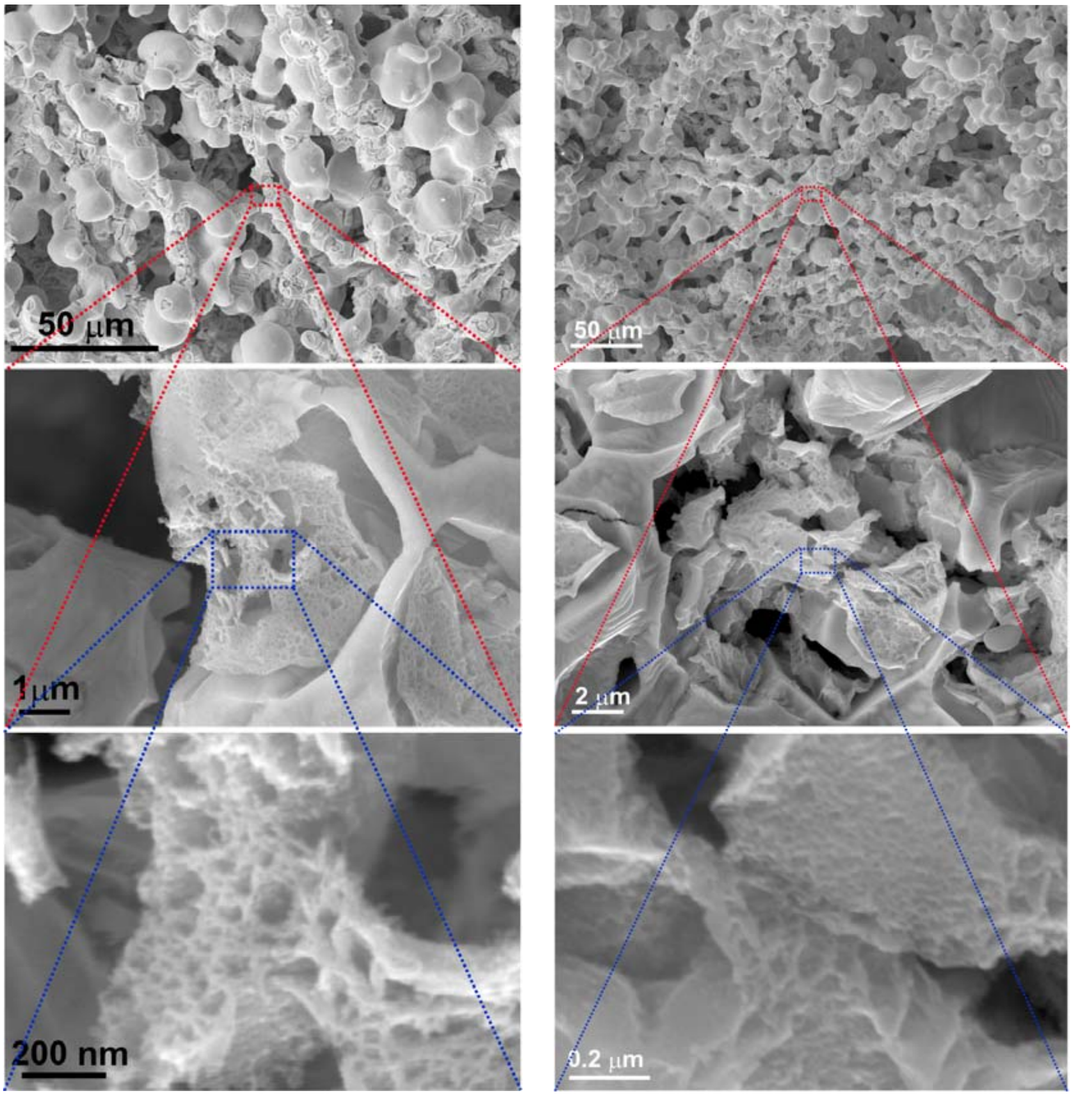

Figure S7. SEM images showing $Ni_{30}Mn_{70}$ microporous nanostructure following dealloying from two different areas.

10. XPS analysis of hierarchical nanoporous Ni chemical bonding states

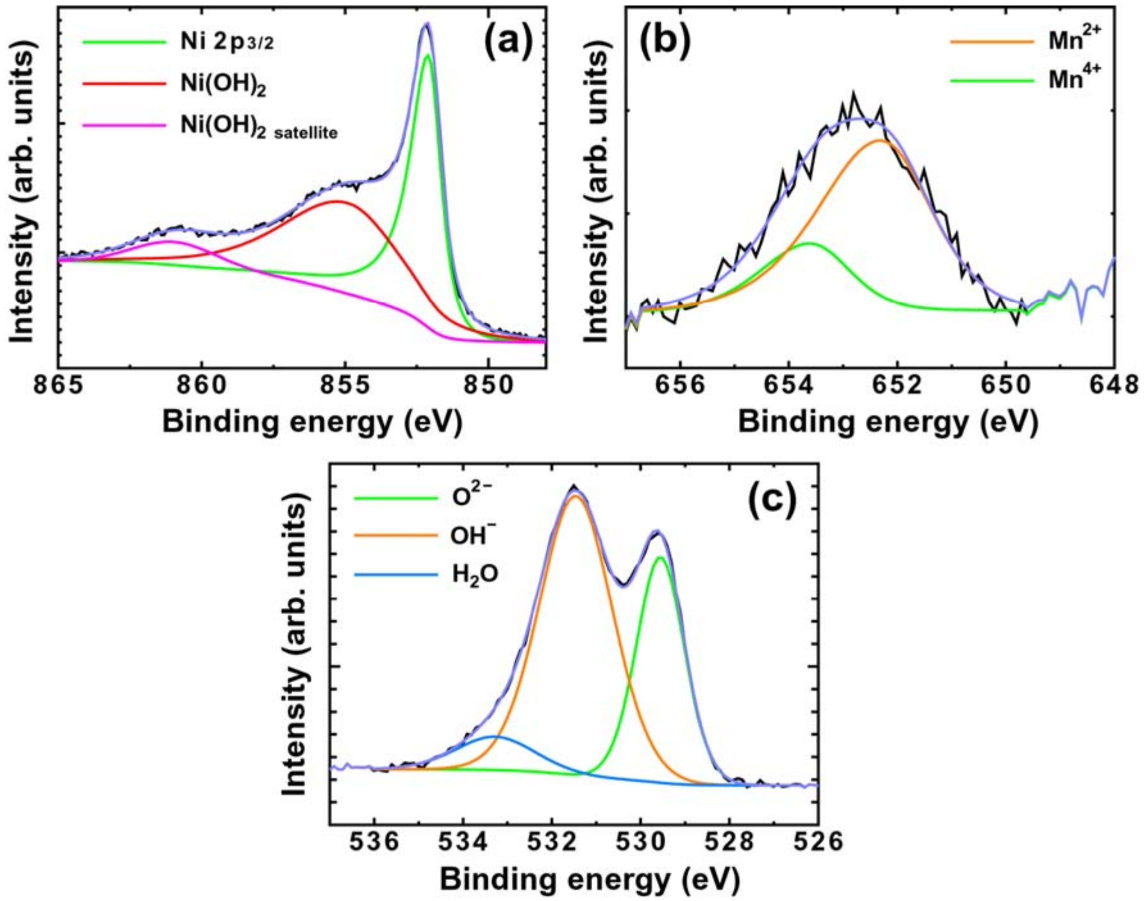

Figure S8. XPS spectra of (a) Ni 2p, (b) Mn 2p, and (c) O 1s orbits for hierarchical nanoporous Ni after dealloying. The metal cations consist of divalent Mn ions, tetravalent Mn ions, and divalent Ni ions.

11. Nanopore size distribution

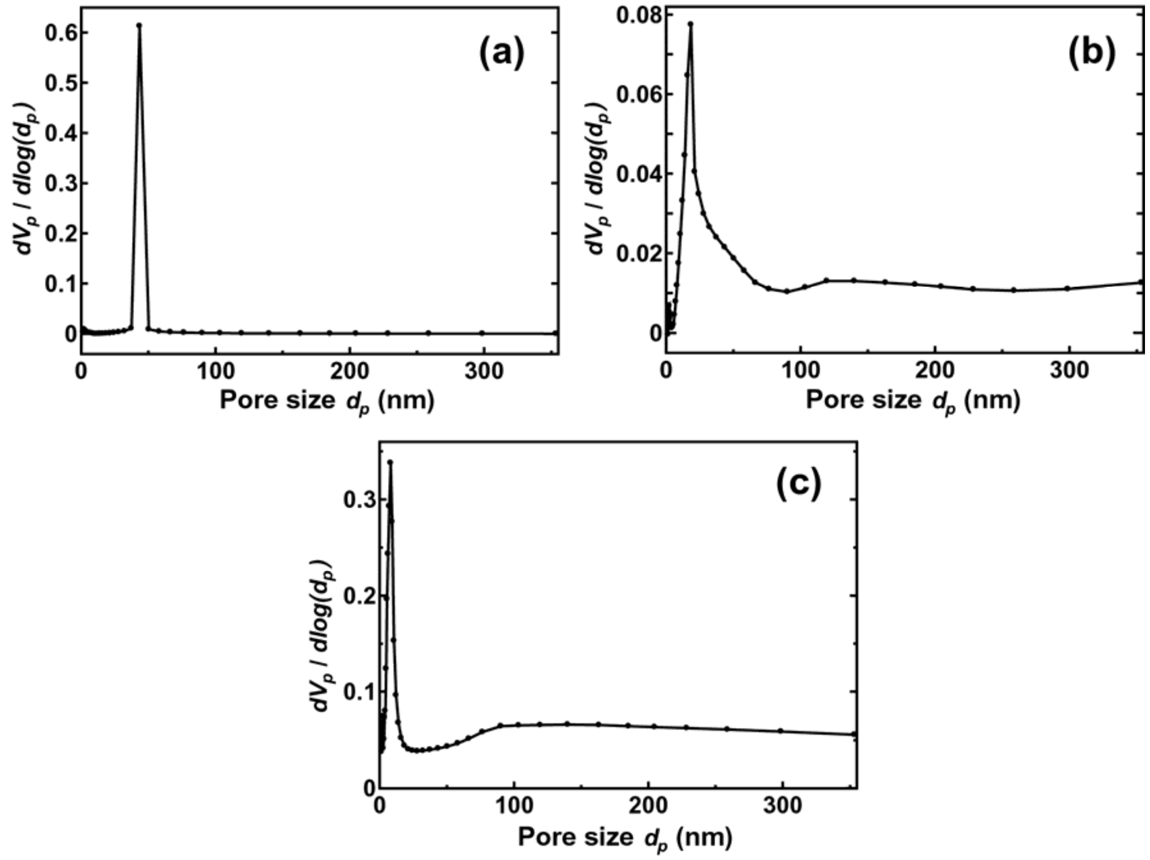

Figure S9. Nanopore size distributions from BJH method for (a) bi-modal and (b) tri-modal NPG, and for (c) dealloyed hierarchical nanoporous Ni.

12. Micropore size distribution

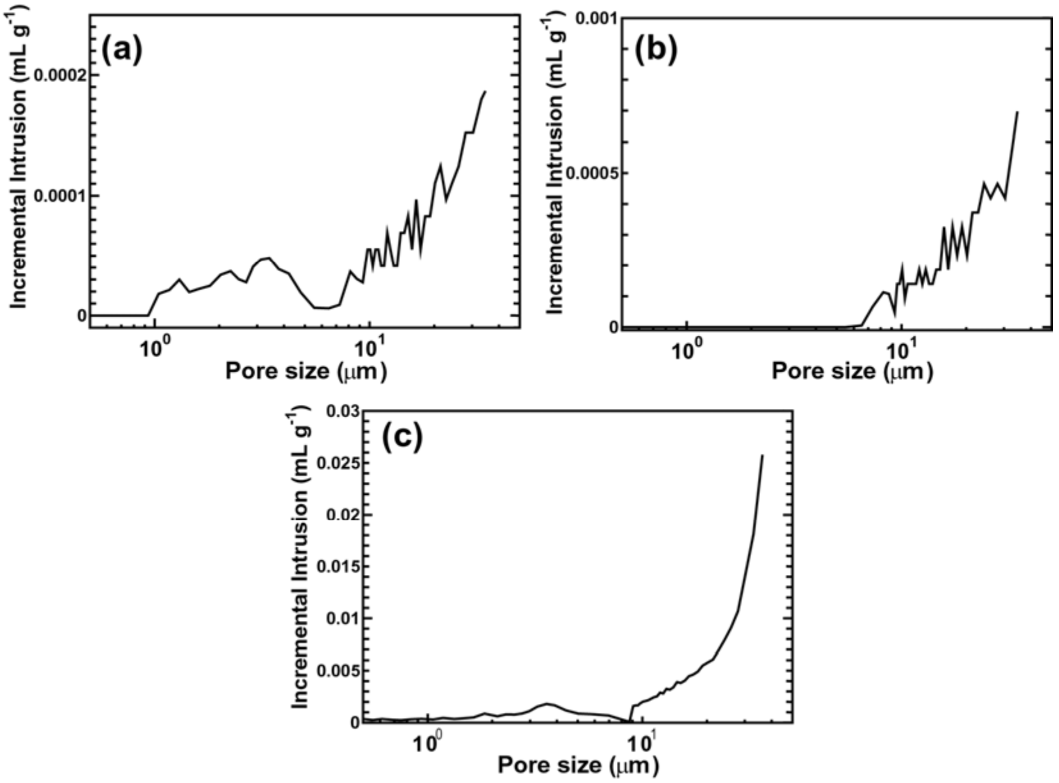

Figure S10. Micropore size distributions from mercury porosimetry analysis technique for (a) bi-modal and (b) tri-modal NPG, and for (c) dealloyed hierarchical nanoporous Ni.

13. Electronic conductance measurements

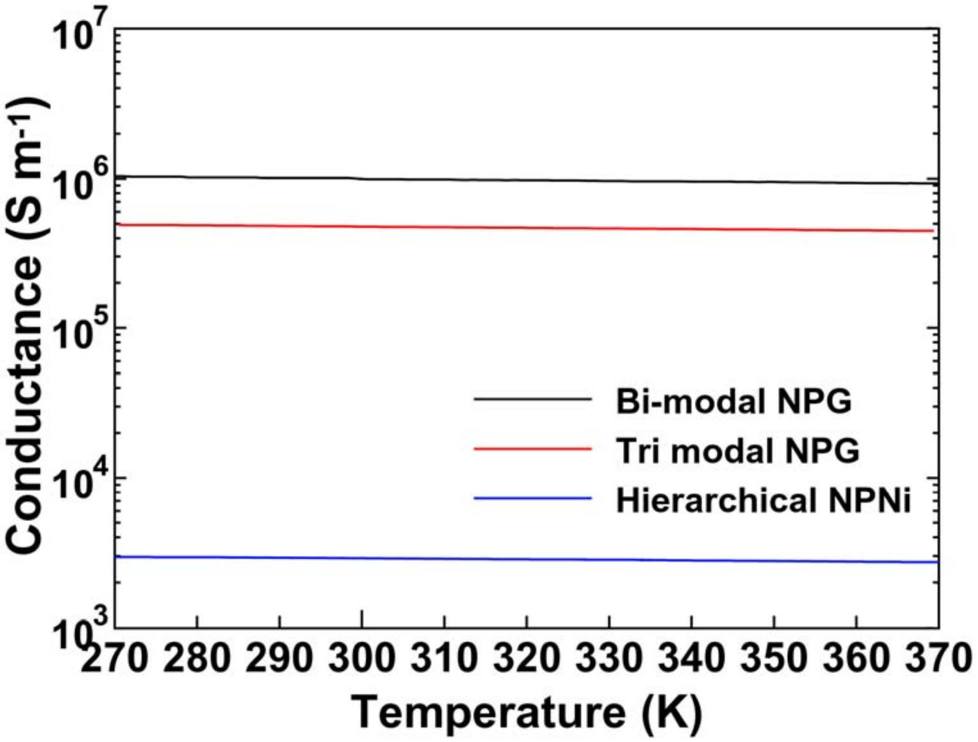

Figure S11. Electronic conductance in 270–370-K range for bi-modal and tri-modal nanoporous Au (NPG), and for dealloyed hierarchical nanoporous Ni (NPNi).

14. Electrochemical impedance measurement for hierarchical nanoporous Ni

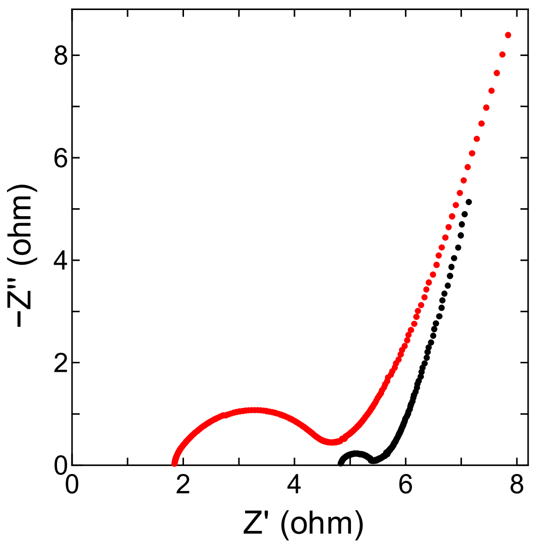

Fig. S12. Nyquist plot of hierarchical nanoporous Ni electrode tested from 100 kHz to 0.1 Hz at 50-mV amplitude before (black) and after (red) stability testing for 2000 cycles. The change in the Nyquist plot is attributed to the change in the electrode nanostructure during the cycling test.

15. Microstructure and EELS chemical mapping of hierarchical nanoporous Ni after cyclic test

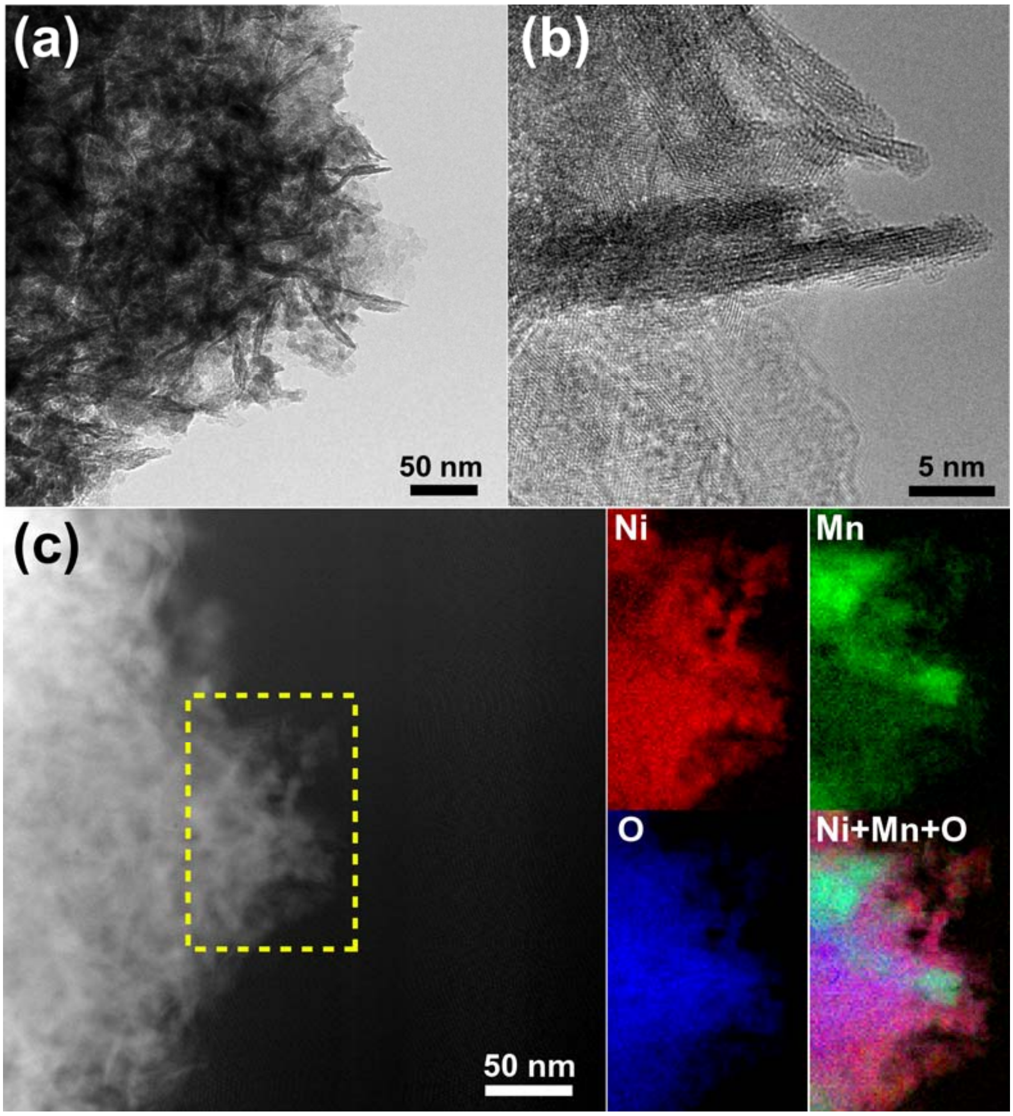

Figure S13. TEM image and EELS chemical map of hierarchical nanoporous Ni after cyclic test. (a) Low- and (b) high-magnification images showing the rod-like nanostructures created on the surface. (c) STEM image and chemical maps of the selected area showing the distribution of Ni (red), Mn (green), and O (blue).The reduction in MnO_x content is clearly confirmed by the EELS mapping, in comparison with Fig. 3.

References

[S1] Y. Kanoko, K. Ameyama, S. Tanaka, B. Hefler, *Powder Metall.* **2014**, *57*, 168.

[S2] J. P. Liu, J. Jiang, C. W. Cheng, H. X. Li, J. X. Zhang, H. Gong, H. J. Fan, *Adv. Mater.* **2011**, *23*, 2076.
